# Girdling behavior of the longhorn beetle modulates the host plant to enhance larval performance

**DOI:** 10.1186/s12862-024-02228-z

**Published:** 2024-04-18

**Authors:** Min-Soo Choi, Juhee Lee, Jeong-Min Kim, Sang-Gyu Kim, Youngsung Joo

**Affiliations:** 1 School of Biological Sciences, Seoul National University, 00826 Seoul, Republic of Korea; 2 Department of Biological Sciences, Korea Advanced Institute of Science and Technology, 34141 Daejeon, Republic of Korea; 3 Department of Biological Sciences and Biotechnology, Chungbuk National University, 28644 Cheongju, Republic of Korea

**Keywords:** Herbivory, Insect offense, Behavioral modulation, Plant-mediated herbivore interaction, *Phytoecia Rufiventris*

## Abstract

**Background:**

Preingestive behavioral modulations of herbivorous insects on the host plant are abundant over insect taxa. Those behaviors are suspected to have functions such as deactivation of host plant defenses, nutrient accumulation, or modulating plant-mediated herbivore interactions. To understand the functional consequence of behavioral modulation of insect herbivore, we studied the girdling behavior of *Phytoecia rufiventris* Gautier (Lamiinae; Cerambycidae) on its host plant *Erigeron annuus* L. (Asteraceae) that is performed before endophytic oviposition in the stem.

**Results:**

The girdling behavior significantly increased the larval performance in both field monitoring and lab experiment. The upper part of the girdled stem exhibited lack of jasmonic acid induction upon larval attack, lowered protease inhibitor activity, and accumulated sugars and amino acids in compared to non-girdled stem. The girdling behavior had no effect on the larval performance of a non-girdling longhorn beetle *Agapanthia amurensis*, which also feeds on the stem of *E. annuus* during larval phase. However, the girdling behavior decreased the preference of *A. amurensis* females for oviposition, which enabled *P. rufiventris* larvae to avoid competition with *A. amurensis* larvae.

**Conclusions:**

In conclusion, the girdling behavior modulates plant physiology and morphology to provide a modulated food source for larva and hide it from the competitor. Our study implies that the insect behavior modulations can have multiple functions, providing insights into adaptation of insect behavior in context of plant-herbivore interaction.

**Supplementary Information:**

The online version contains supplementary material available at 10.1186/s12862-024-02228-z.

## Background

Coevolutionary theories predict that plant defense and insect offense exert selective pressure that favors diversification of each other [1]. Central to understanding the arms race between plants and insect herbivores have been the phytochemistry and the molecular adaptation of insects to it [2, 3]. However, the chemical conflict occurs at the latest stage of herbivory, only after the ingestion occurs. Before ingestion, an interesting class of insect behavior is observed in many clades: behavioral modulation. Vein cutters cut the veins of the leaf, trenchers cut a line through the leaf, and girdlers chew around the petiole or stem [4]. These behavioral modulations are found in various insect taxa of Lepidoptera [5], Coleoptera [6, 7], Hemiptera [8], and Orthoptera [9]. The convergent evolution of similar behavioral modulations from distant taxa indicates it is an adaptation.

One well-characterized function of the behavioral modulation is the neutralization of plant defense, especially canal-borne exudates that are blocked by vein cutting and trenching [6, 10]. However, though behavioral modulation is abundantly conducted on plants without secretory canals, their functional consequences in plant-herbivore interaction is rarely tested. Particularly, only a few studies have proposed the potential role of the girdling behavior as exposure of vasculature to apply saliva [11] or nutrient accumulation at the girdled part [7, 8] and the downstream effects on insect performance are not known, raising questions on the functional consequence of girdling behavior.

Herbivorous insects largely depend on the quality of the host plants [12]; the dependency suggests that examination of the effect of the girdling behavior on the metabolic and defensive traits of plants is required to assess the functional consequences of the behavior. Insect attack has significant effects on the nutrient status of plants and the effect of modified plant chemistry on insect performance is diverse [13]. Moreover, insect herbivore faces plant defenses comprising unpalatable substances (e.g., secondary metabolites [14], proteinase inhibitor [15], and hardened cell walls [16]), and natural enemies of herbivores [17]. A substantial part of plant defense is activated by attack primarily through jasmonic acid (JA) signaling [18], which is referred induced defense.

Different herbivores feeding on the same plant interact with each other via plant traits including systemic responses [19] and altered plant appearance [10]. As behavioral modulation changes plant morphology [20] and palatability [21], it may alter the responses of other herbivores sharing the same host plant. Indeed, canal cutting promotes the feeding of other herbivores that originally do not feed on plants with secretory canals [21]. However, it is not known whether the effect of behavioral modulation on other herbivores are beneficial to the modulating insects.

To systematically assess the functional consequence of behavioral modulation of insect herbivores, we studied a girdling longhorn beetle *Phytoecia rufiventris* Gautier (Lamiinae; Cerambycidae) and a non-girdling longhorn beetle *Agapanthia amurensis* Kraatz (Lamiinae; Cerambycidae) which both oviposit on an introduced species *Erigeron annuus* L. (Asteraceae) in South Korea. The *P. rufiventris* female girdles around the stem of Asteraceae host plants including *E. annuus* before oviposition (Supplementary Video S1) while *A. amurensis* female lays an egg inside the stem without girdling. To determine the functional consequences of the girdling behavior using experimental girdling, we tested the following three hypotheses: (1) The girdling behavior of female *P. rufiventris* facilitates larval growth inside the girdled stem, (2) The girdling behavior impairs the plant resistance and enhances nutrients in the girdled stem, and (3) The appearance of the girdled stem is decreased to a competing herbivore.

## Results

### Two longhorn beetles sharing *Erigeron annuus* as the host plant show distinct natural histories

**Fig. 1:**
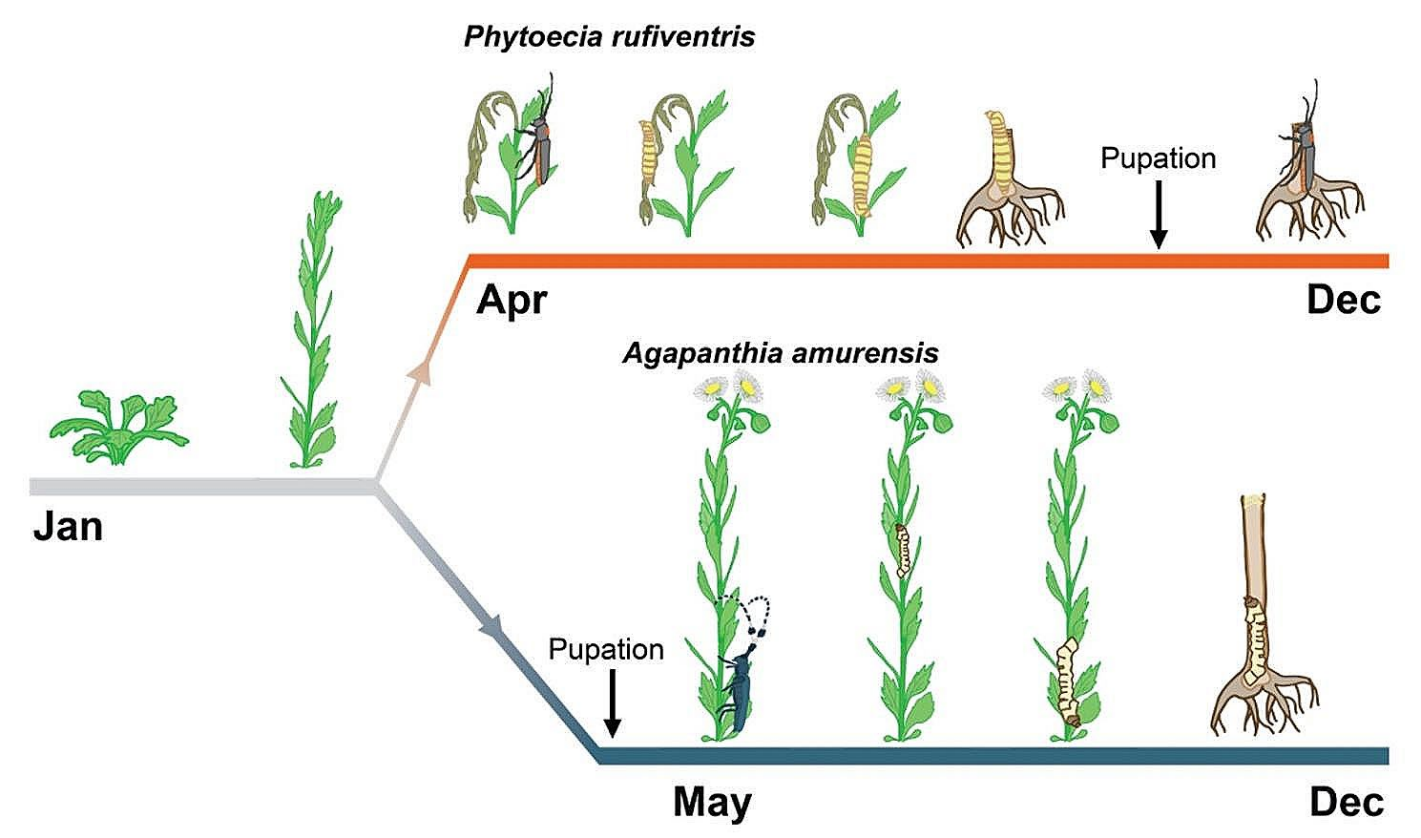
Natural history of *Erigeron annuus* and two longhorn beetles, *Phytoecia rufiventris* and *Agapanthia amurensis*. Adult emergence and oviposition timing in field observation sites are indicated as the adult. The larval feeding pattern is indicated as the larval position inside the stem

We observed the natural history of two Lamiinae longhorn beetles, *Phytoecia rufiventris* and *Agapanthia amurensis*, by monitoring *Erigeron annuus* (Asteraceae) as a shared host plant of two beetles in our field sites in Korea (Fig. 1, Supplementary Table S1). *Phytoecia rufiventris* laid eggs in *E. annuus* predominately while they also laid eggs in *Artemisia princeps. Erigeron annuus* bolted in April and *P. rufiventris* adults emerged from mid-April to July, girdled and laid eggs inside the stem of *E. annuus* plants. Every *E. annuus* stem with a feeding pattern at the lower part also had a feeding pattern at the upper part but not vice versa i.e., the larvae first fed on the upper part of the girdled stem and then move down beyond the girdles. In September, the larva cut the basal part of the stem and pupated inside the root-shoot junction. The adult overwintered inside the stem until subsequent April.

In contrast to *P. rufiventris*, *A. amurensis* overwintered as a larva and pupated during April. The adult *A. amurensis* thus emerged from May to July and laid eggs at the lower part of *E. annuus* stem without girdling. The hatched larva first moved and fed towards the shoot apex, then moved down to the lower part of the stem and cut in the middle of the dead stem. The larva stayed inside the stem until subsequent April.

### Girdling behavior of *Phytoecia rufiventris* cuts all vascular bundles to induce cell death at the upper part of the *E. annuus* stem

**Fig. 2:**
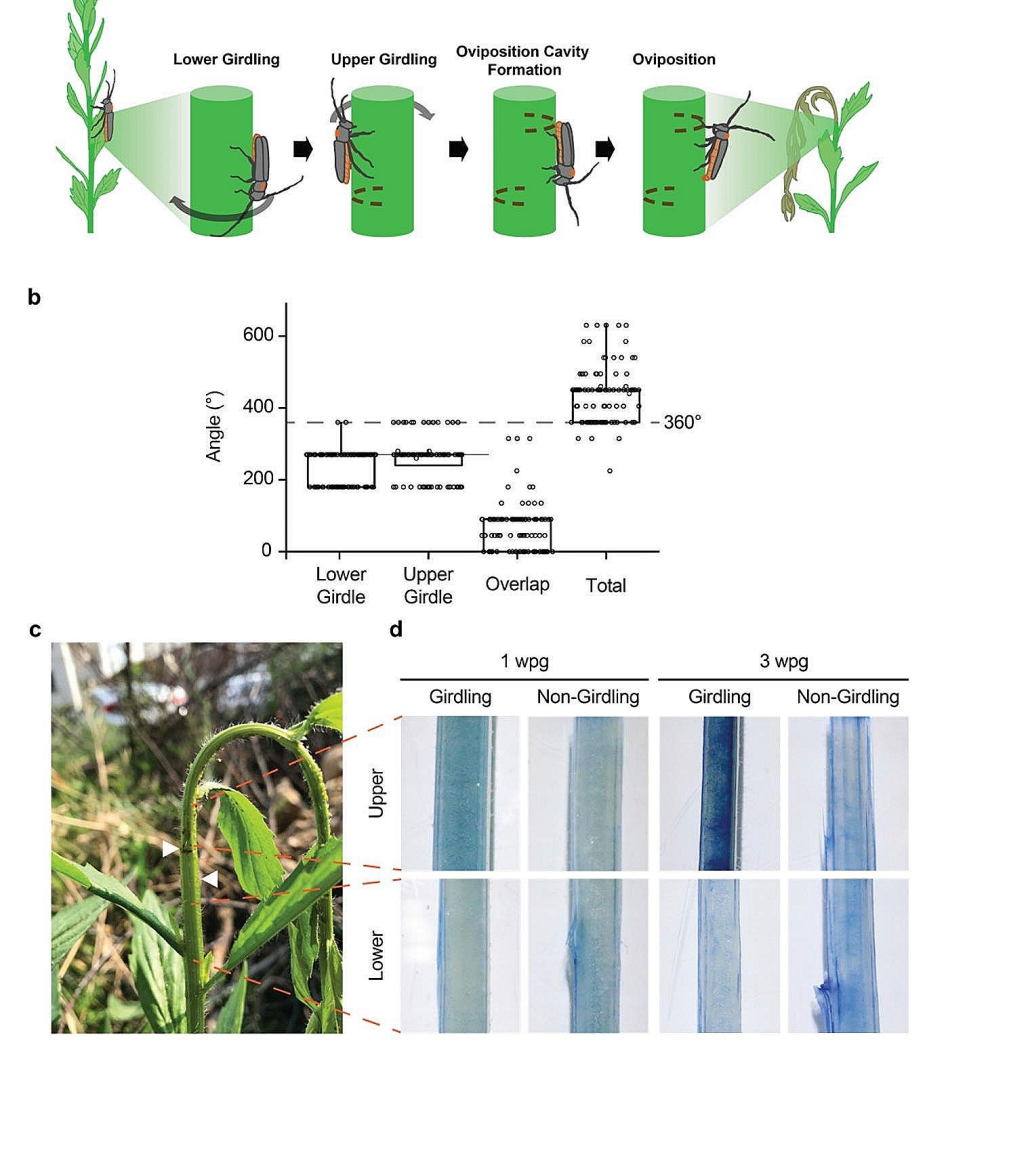
Observations on girdling behavior of *Phytoecia rufiventris*. (**a**) Behavioral sequence of girdling and oviposition of *P. rufiventris*. (**b**) Girdle angles of lower and upper girdling (*N* = 100). Box indicates 1st and 3rd quantiles. (**c**) Morphology of naturally girdled *E. annuus.* Two girdles are indicated with white triangles. (**d**) Trypan-blue staining of the experimentally girdled and non-girdled stem of *E. annuus* at 1 and 3 wpg. Blue staining indicates cell death

To examine the functional consequence of girdling behavior of *P. rufiventris*, we first characterized the girdling behavior (Fig. 2a and b, Supplementary Video S1, approximately 6 min). *Phytoecia rufiventris* female girdles two arcs (lower and upper girdles) with about 1 cm apart. The female makes an oviposition cavity by chewing the epidermis and then lays an egg inside the stem. The upper part of the girdled *E. annuus* plant lost its turgor within an hour and showed dropping morphology (Fig. 2c, Supplementary Video S2, approximately 9 min). The girdles divided the stem into the upper (14.76 ± 4.07 cm) and lower part (50.72 ± 16.13 cm) respectively (*N* = 73 and 61, respectively). Though neither of the lower and upper girdles was a full circle, they together cut every vascular bundle (Fig. 2b). Two semicircle artificial girdles mimicked the natural girdling without fracture of the upper part (Supplementary Fig. S1). The upper part of the girdled stem underwent cell death within 1-week post girdling (wpg) while the lower part of the girdled stem remained viable (Fig. 2d). The cell death in the upper part of the girdled stem became more severe at 3 wpg, as shown by stringer Trypan-blue staining at the upper part of the girdled stem.

### Larval performance of *P. rufiventris* is enhanced by girdling behavior

**Fig. 3:**
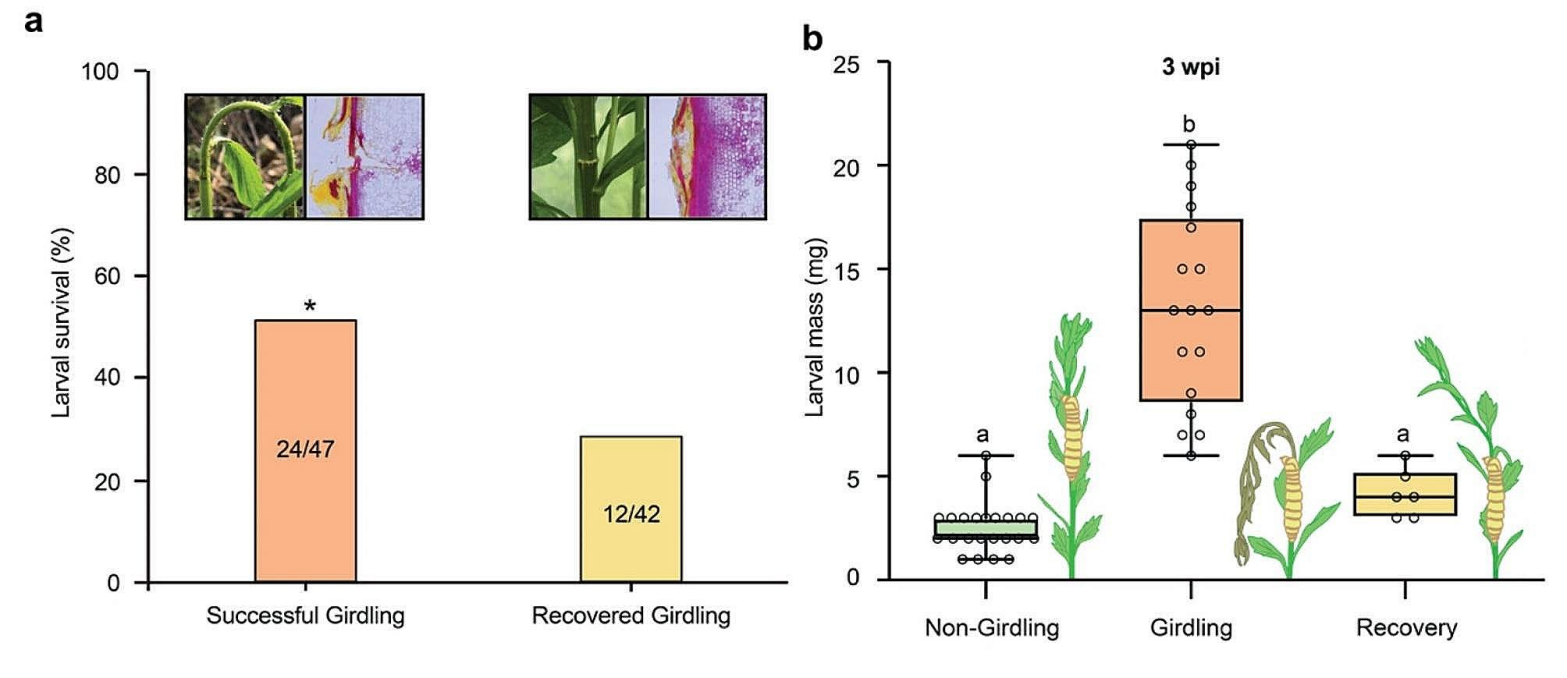
Effect of girdling behavior on the larval performance of *P. rufiventris*. (**a**) Larval survival rates in successfully girdled (*N* = 47) and recovered (*N* = 42) *E. annuus* plants measured in naturally girdled plants (* *P* < 0.05, Chi-squared test). The left images represent the morphology of girdled and recovered *E. annuus* plants while the right images represent Phloroglucinol staining of the longitudinal section of girdled and recovered part. (**b**) Larval mass measured from the experimentally girdled and non-girdled *E. annuus* plants. Boxes indicate the 1st and 3rd quantiles (significant differences are indicated as different letters; *P* < 0.05, One-way ANOVA followed by Tukey’s HSD)

We found the girdled *E. annuus* occasionally reconnected the damaged vascular bundles and restored the turgor to the upper part in field observation. Phloroglucinol staining of the longitudinal section of experimentally girdled and recovered stem showed reconnection of the xylem in recovered *E. annuus* plants (Fig. 3a). In such recovered stems, larval survival of *P. rufiventris* was significantly lower than in successfully girdled *E.* annuus stem (Fig. 3a).

We then mimicked the girdling behavior and inoculated *P. rufiventris* eggs in the *E. annuus* stem (Supplementary Fig. S1a). At three wpg, the larval mass was nearly five times higher in girdled plants than in non-girdled plants (Fig. 3b). As in the field monitoring, the experimentally girdled stems occasionally recovered (Supplementary Fig. S1b). The larvae in such recovered stems showed a similar mass to the larvae in non-girdled plants (Fig. 3b).

### The upper part of the girdled stem is modulated into a better food source

**Fig. 4:**
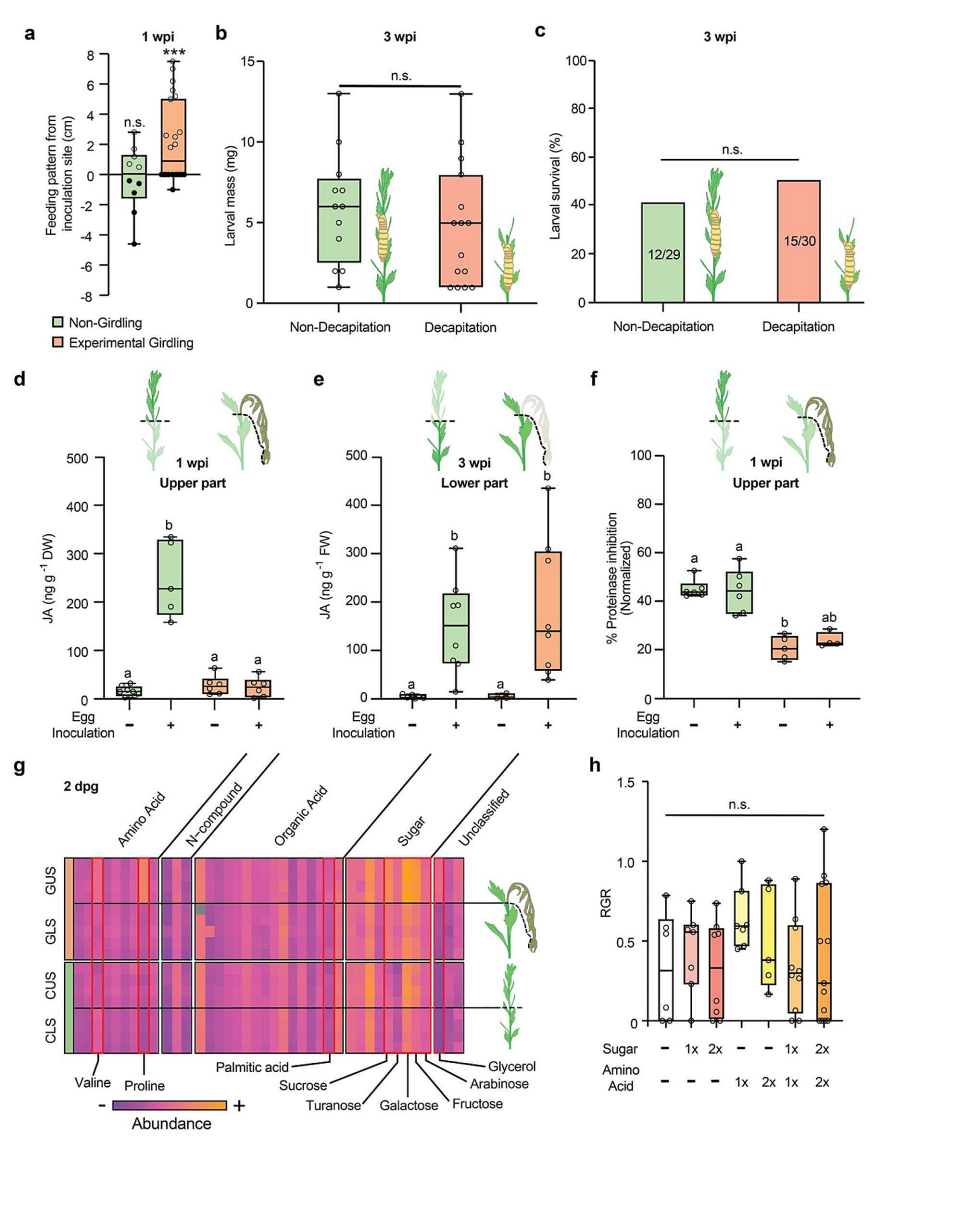
The upper part of the *E. annuus* plant is modulated by girdling behavior. (**a**) Feeding patterns of *P. rufiventris* larva between the upper and lower parts of the stem one week after egg inoculation. Empty dots indicate the upper limit of the feeding pattern, whereas filled dots indicate the lower limit of the feeding pattern. Boxes indicate the 1st and 3rd quantiles. Statistic comparisons were performed between the upper and lower limits in the same treatment groups. (n.s., no significant difference; *** *P* < 0.001, Student’s T-test). (**b-c**) Larval performance in decapitated plants, with (**b**) larval mass and (n.s., no significant difference, Chi-squared test) (**c**) larval survival rates (n.s., no significant difference, Student’s T-test). (**d-e**) Jasmonic acid levels were measured with and without experimental girdling and egg inoculation at (**d**) the upper part and € the lower part of the stem. (Significant differences are indicated as different letters; *P* < 0.05, One-way ANOVA followed by Tukey’s HSD). (**f**) Normalized % proteinase inhibition in experimentally girdled and non-girdled stems (significant differences are indicated as different alphabet letters; *P* < 0.05, Kruskal-Wallis’ test followed by Dunn’s test). (**g**) Metabolic profiles of the upper and lower stems of *E. annuus* with and without experimental girdling. Metabolites that accumulated only at the upper part of the girdled stem are indicated with the red box and their compound names (*P* < 0.05, One-way ANOVA followed by Tukey’s HSD). The abundance values were log-transformed for plotting. GUS, girdled upper stem; GLS, girdled lower stem; CUS, control upper stem; CLS, control lower stem

As in our field observation, the larva preferentially fed on the upper part during the first week after egg inoculation whereas larvae in non-girdled stems showed no feeding preference (Fig. 4a). Moreover, larval survival and mass were not facilitated when the upper part of the stem was removed by decapitation (Fig. 4b and c). Thus, we suspected that the upper part of the girdled *E. annuus* stem is modulated into a better food source for the larva.

To understand the mechanistic background of the enhanced larval performance in girdled plants, we compared phytohormonal responses of the upper and lower part of experimentally girdled and non-girdled plants. First, we found that while the upper part of non-girdled plants showed increased JA levels upon larval attack, the upper part of girdled plants was not able to increase JA levels during the first week of larval feeding (Fig. 4d). This impaired defense in the girdled *E. annuus* was only present in the upper part of the stem while the lower parts of the stem showed increased JA levels regardless of experimental girdling, 3 weeks after the egg inoculation (Fig. 4e). In contrast, decapitated stems showed increased JA levels upon larval attack (Supplementary Fig. S2), highlighting importance of the upper apart of the girdled stem in facilitating larval growth. While salicylic acid (SA) levels at the upper part of the stem were not affected by both experimental girdling and egg inoculation (Supplementary Fig. S3a), abscisic acid (ABA) levels were significantly increased by experimental girdling in the same region (Supplementary Fig. S3b). Egg inoculation had no significant effect on ABA levels in both girdled and non-girdled *E. annuus* plants.

Additionally, we measured the proteinase inhibitor activity of girdled and non-girdled stems to assess the palatability of the girdled tissues (Fig. 4f). The proteinase inhibitor activity was not induced by the larval attack but it was reduced by experimental girdling. The protease inhibitor activity was marginally restored in girdled *E. annuus* stem by larval attack but the activity level was not significantly different from not attacked and girdled *E. annuus* stem.

We then tested the nutritional value of the girdled stem by sampling the upper and lower parts of stems with and without experimental girdling 2 days after experimental girdling, when the larval feeding starts. Primary metabolites were analyzed using gas chromatography–mass spectrometry (GC-MS) (Supplementary Table S2). Principal component analysis (PCA) of the 40 identified metabolites showed that the metabolomes of the upper parts were largely altered by experimental girdling, while the lower parts were only affected to a modest degree (Supplementary Fig. S4). We identified nine metabolites that were significantly accumulated in the upper part of the experimentally girdled stems while not accumulated in the upper part of the non-girdled stems (Fig. 4g): palmitic acid, glycerol, valine, proline, sucrose, turanose, galactose, fructose, and arabinose. The accumulation of soluble sugar in the upper part of girdled stems was confirmed by an anthrone assay (Supplementary Fig. S5).

Subsequently, we tested whether the accumulated nutrient sugars and amino acids in the upper part of the stems are sufficient to facilitate larval growth. We reared *P. rufiventris* larvae on semi-artificial diets made of non-girdled stem powder supplemented with sugars and amino acids, corresponding to the previous measurements. The three most abundant sugars (fructose, sucrose, and galactose) and two amino acids (proline and valine) in the upper part of the girdled stems were selected. Unexpectedly, the larval relative growth rate was not significantly altered by sugar and/or amino acid supplementation, even with the double amount of observed difference between the girdled and non-girdled upper part of the stem (Fig. 4h, Supplementary Fig. S6).

### Non-girdling *Agapanthia amurensis* does not benefit from girdling behavior

**Fig. 5:**
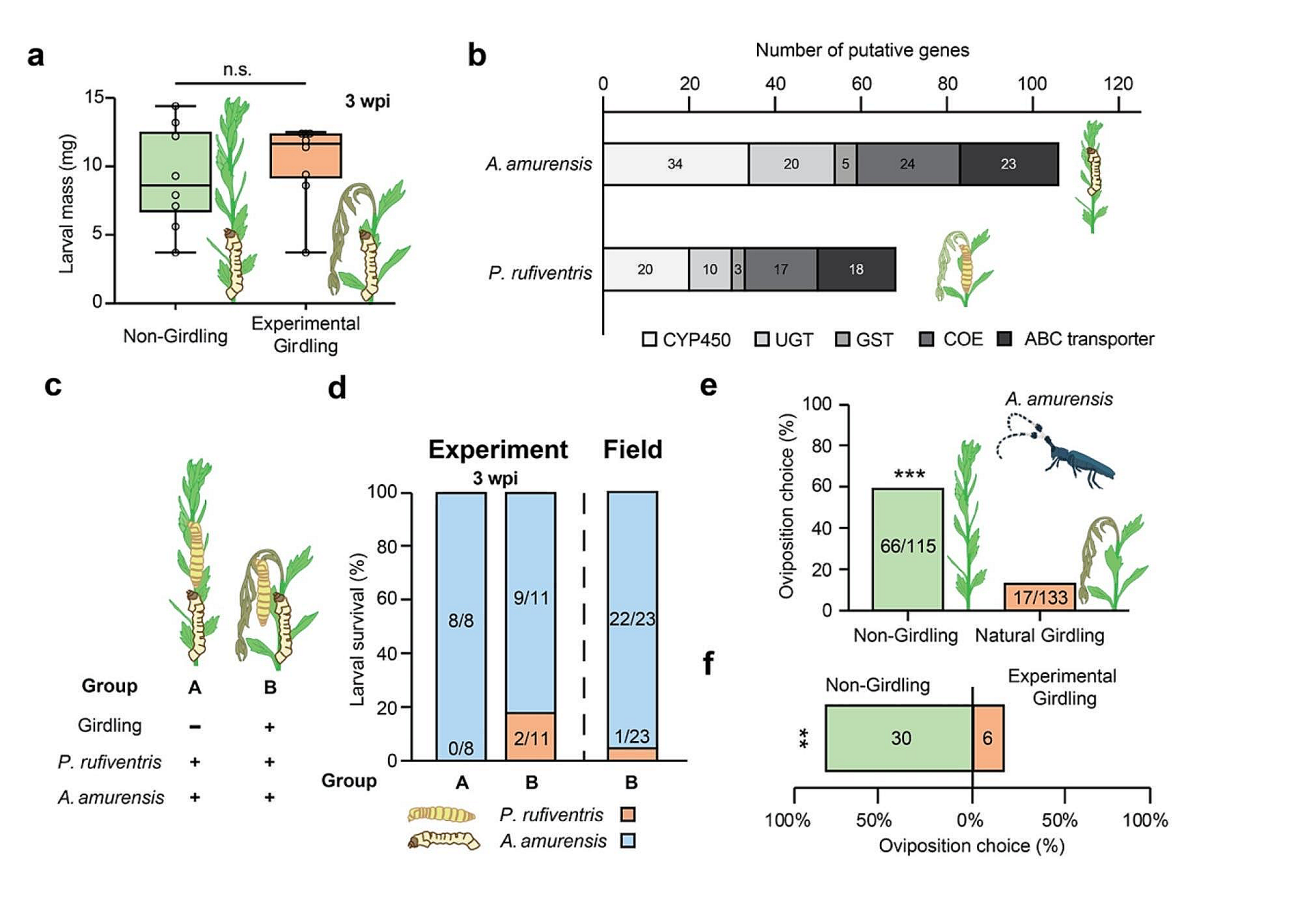
Effect of girdling behavior on *Agapanthia amurensis*. (**a**) Larval mass of *A. amurensis* measured from the egg inoculation experiment. Boxes indicate the 1st and 3rd quantiles (n.s., no significant differences). (**b**) Number of putative detoxification genes identified from *de novo* assembled transcriptome of *P. rufiventris* and *A. amurensis* (CYP450; Cytochrome P450, UGT; UDP-glucosyltransferase, GST; Glutathione-S-transferase, ABC transporter; ATP-binding cassette transporter, COE; Carboxylesterase). (**c**) Experimental scheme of the co-infestation assay. The scientific names of insects indicate egg inoculation. (**d**) Larval survival of *P. rufiventris and A. amurensis* larvae in co-infested E. annuus stem. Treatments for each group are indicated in Fig. 5c (**e-f**) Oviposition preference of *A. amurensis* on girdled and non-girdled *E. annuus* in (**e**) field monitoring (***; *P* < 0.001, Chi-squared test) and (**f**) choice assay (**; *P* < 0.01, Fisher’s exact test). The field-girdled *E. annuus* contained *P. rufiventris* egg inside the stem while experimentally girdled *E. annuus* stem did not

With our experimental evidence on the adaptive value of girdling behavior, we tested whether girdling behavior provides general benefits to a non-girdling longhorn beetle, *A. amurensis*. Unlike in *P. rufiventris*, experimental girdling did not significantly facilitate larval growth of *A. amurensis*, regardless of the presence of *P. rufiventris* larva in the same stem (Fig. 5a).

The asymmetry in the effect of girdling on two species led us to speculate about the differential chemical adaptation of two species to plant metabolites. To test this idea, we investigated the detoxification capacity of two species at the transcriptomic level. We used *de novo* assembled gut transcriptome of two species to extract putative detoxification genes of five families: Cytochrome P450s (CYP450s), UDP-glycosyltransferases (UGTs), Glutathione-S-Transferases (GSTs), Carboxylesterases (COEs), and ABC transporters. Interestingly, the number of putative detoxification genes was higher in the assembled transcriptome of *A. amurensis* than that of *P. rufiventris* for all 5 families of genes investigated (Fig. 5b).

### *Phytoecia rufiventris* avoids competition with *Agapanthia amurensis* via the decreased appearance of girdled stem

We observed co-infestation of *P. rufiventris* and *A. amurensis* on same *E. annuus* plants in the field monitoring. Notably, *A. amurensis* larvae mostly persisted in stems of such plants while *P. rufiventris* larvae were dead (Fig. 5c and d). To test whether competition is sufficient to decrease the larval survival of *P. rufiventris*, we conducted a co-infestation assay (Fig. 5c). Regardless of experimental girdling, in most stems where two longhorn beetles were inoculated together, *A. amurensis* larvae showed priority (Fig. 5d).

To test whether the girdled *E. annuus* plants are hidden from the oviposition choice of *A. amurensis*, we measured the oviposition rate of *A. amurensis* on naturally girdled and non-girdled *E. annuus* plants in field sites. The proportion of plants chosen for *A. amurensis* oviposition was higher in non-girdled *E. annuus* plants than in girdled plants (Fig. 5e). To confirm the decreased appearance of girdled *E. annuus* plants to *A. amurensis*, we conducted an oviposition choice assay of *A. amurensis* on girdled and non-girdled *E. annuus* plants. The female *A. amurensis* significantly preferred non-girdled *E. annuus* plants for oviposition over girdled *E. annuus* plants (Fig. 5f).

## Discussion

Natural history observations on behavioral modulations suggest various functions of behavioral modulations. As most behavioral modulations damage the vascular bundles [9], the subsequent effects have been predicted to be the inhibition of defense signaling [20] and accumulation of nutrients [22, 23]. Moreover, the modified morphology of modulated plants is suspected to be utilized for evasion of natural enemies [20]. However, other than disarming defense mediated by canal-borne exudates, experimental testing on functional consequences of behavioral modulations at the molecular level has been seldom compared to copious hypotheses.

In consistent to the cell death observed at the upper part of the girdled stem, JA induction and proteinase inhibitor activity were decreased at the region. Inhibition of JA signaling is strong evidence of inhibited induced defense against chewing herbivores [24]. Although the inhibition of defenses was restricted to the upper part of the stem, the girdling-dependent feeding preference on the upper part enabled the larvae to selectively consume the modulated food source during the early stages. The susceptible region provided could boost the growth of vulnerable early larval stages and face the induced defense of the lower part of the stem with a better capacity to tolerate plant defense. The inhibition of plant defense at the physiological level is achieved by various insect strategies including symbionts [25] and effectors [26]. We propose that the plant tissue which is freshly killed by the girdling behavior also exhibit inhibited defense.

In addition to deactivated defenses, accumulation of nutrients also occurred in the upper part of the girdled stem, as in girdling of alfalfa hopper and twig girdler [7, 8]. However, the nutrient, especially soluble sugar, can be negatively correlated with insect performance [13]. In our study, the nutrient supplementation in an artificial diet had no significant effect solely on larval growth. Nevertheless, the joint effect of accumulated nutrients and impaired defense in girdled *E. annuus* stem remains to be studied.

Interestingly, experimental girdling had no significant effect on the growth of *A. amurensis* larvae. In studies performed using generalists to test the effect of trenching, the behavioral modulation was a prerequisite for feeding but not always sufficient [27]. As *A. amurensis* is capable of feeding on *E. annuus* without girdling behavior, possible explanation are that *A. amurensis* may not induce a strong defense response in *E. annuus* or cope well with the defenses. To test the latter possibility, we mined the transcriptomes of *P. rufiventris* and *A. amurensis* and compared the number of putative detoxification genes, as the gene duplication events are thought to be favored by pressure exerted by plant substances [28, 29]. We interrogated the enzymes that are generally but not exclusively associated to plant metabolite detoxification as the specialized detoxification mechanisms of *P. rufiventris* and *A. amurensis* are not known. Insects detoxify toxic metabolites by cleavage (e.g., Carboxylesterase; COE [30]), oxidation (e.g., Cytochrome P450; CYP450 [31]), and the addition of moiety (e.g., Glutathione-S-Transferase; GST [32], UDP-Glycolic Transferase; UGT [33]). Moreover, plant metabolites are rapidly transported by transporters such as ATP-binding cassette (ABC) transporters [34]. The higher putative detoxification gene numbers of *A. amurensis* in compared *to P. rufiventris* might be associated with their ability to digest plant metabolites.

Nevertheless, our current analyses do not include identification of bioactive resistive compound produced by *E. annuus* and deactivated by the girdling behavior, leaving an interesting topic for further study. *Erigeron annuus* synthesizes a variety of secondary metabolites including terpenoids, flavonoids, organic acid glycosides, and polyacetylenes [35–38]. The bioactivities of plant secondary metabolites are highly context-dependent; indeed, untargeted metabolomics coupled with bioassays are further required to the chemical interaction between *E. annuus* and *P. rufiventris*, along with the effect of behavioral modulation on plant secondary metabolism.

The drooping stem is a widespread phenotype in herbaceous plants that decreases appearance to herbivores [20]. The girdling-induced drooping of *E. annuus* decreased the appearance to *A. amurensis* which is a harmful competitor of *P. rufiventris*. Although co-infestation of multiple herbivores on a plant can be beneficial [39] or neutral [40] for herbivores, restricted unidirectional movement of larvae inside the stem causes unavoidable competition between *P. rufiventris* and *A. amurensis*. This indirect effect of girdling behavior, evading a competitor, is a novel function of behavioral modulation, which we propose is an exaptation of counteradaptation to plant defense. Rodent herbivores also modulate the physical properties of plants to decrease their appearance, implying that disguising through plant modulation has a general adaptive value across animals [41].

## Conclusion

In conclusion, we demonstrated that the girdling behavior of *P. rufiventris* decreases host plant defense and increases nutrients. The girdling behavior also decreases the risk of harmful competition with *A. amurensis* via the lowered appearance of the girdled plants. Insect herbivores have developed various array of offensive strategies to deal with plant defense [42]. We conclude that the behavioral modulation on plants can have manipulating effects on host plant physiology and morphology, which clearly are adaptive for insect herbivores.

## Methods

### Plant growth and insect rearing

Adult *Phytoecia rufiventris* beetles were collected from our field sites (Supplementary Table S1) between April and July 2019–2022. The adult beetles were maintained at 25–27 °C in transparent acrylic cages (40 × 40 × 40 cm^3^) with *Erigeron annuus* plants under long-day (16 L:8D) conditions. Eggs were collected every three days and inoculated inside the *E. annuus* stem following the previously described method [43] with experimental girdling. The larvae were maintained in *E. annuus* stems in the lab until the basal part of the stem was cut by the larvae, then the larvae were collected from the stems and moved to the short-day condition (12 L:12D) at 25 °C to induce pupation [44]. To break adult sexual diapause, the adults were placed at 0 °C for 16 weeks.

Adult *A. amurensis* adults were collected from the field sites (Supplementary Table S1) from May to July 2019–2022 and reared under the same conditions as those of *P. rufiventris*. The collected eggs were inoculated inside the *E. annuus* stem without experimental girdling and reared inside the stem until the stem is cut by larvae then collected and placed at 0 °C for 16 weeks to induce pupation.

We collected *E. annuus* seeds from a single individual at the Daejeon_1 site (Supplementary Table S1) in July 2019 and dried them with silica gel for 3 weeks. Seeds were germinated a 9 cm x 9 cm x 9 cm pot and were grown under long-day conditions (16 L:8D), 26 °C.

### Field monitoring

Field monitoring was conducted from 2019 to 2022 at field sites in Daejeon, Cheongju, and Jeungpyeong, Korea (Supplementary Table S1). The angles of the girdled scars were measured by visually classifying the angle of the girdle arc by 45°. The positions of the girdles were measured from the ground to the lower girdle and from the shoot apical meristem to the upper girdle. The survival of *P. rufiventris* larvae in successfully girdled and recovered *E. annuus* stems was investigated. The oviposition of *A. amurensis* on girdled and non-girdled *E. annuus* plants was measured by checking the oviposition cavity. The larval survival of *P. rufiventris* in girdled *E. annuus*, with and without *A. amurensis* competition was measured by randomly dissecting naturally girdled *E. annuus* plants.

### Egg inoculation and experimental girdling

Experimental girdling was performed by cutting the vascular bundles of *E. annuus* stems into two lines 1 cm apart, each forming a semicircle. Decapitation was performed by cutting the stem at the corresponding height. *Phytoecia rufiventris* eggs were inoculated between two girdles by digging a scar with a needle and gently inserting it with forceps, as described previously [43]. The egg inoculation was performed right below the cut stem in decapitated *E. annuus* plants. Experimental girdling and egg inoculation were performed 8 weeks after the germination of *E. annuus*. We sampled the upper part of the girdled stem one wpg (weeks post girdling) while the lower part was sampled three wpg, when the actual larval attack was occurring at each part. To enable sufficient growth of larvae for measurement, larval growth was measured in three wpg.

### Trypan blue staining

The *E. annuus* stems were longitudinally cut, stained in 0.4% Trypan blue (aq) for 5 min, and destained with 100% ethanol overnight.

### Primary metabolites and total soluble sugar measurement

The upper and lower parts (each 15 cm) of the girdled stem were collected 2 days after experimental girdling. Samples were freeze-dried and ground by mortar and pestle.

The metabolites in each sample were analyzed as described previously [45]. Briefly, 10 mg of each freeze-dried sample was used for extraction with 1 ml of methanol and the solvent was evaporated by applying a nitrogen flow rate of 0.625 L/min for 5 min. To induce oximation, 30 µL of 20 mg/mL methoxyamine hydrochloride in pyridine, 50 µL of BSTFA (*N, O*-Bis (trimethylsilyl)trifluoroacetamide) with 1% TMCS (chlorotrimethylsilane), 30 µL of 300 µg/mL 2-chloronaphtalene in pyridine was added. 10 µL of 300 µg/mL 2-chloronaphtalene in pyridine for internal standards, was added. The samples were incubated at 65 °C for 60 min. One microliter of the 1/100 diluted sample was injected into the Rtx-5MS column at an injection temperature of 250 °C. A GCMS-QP2020 system (Shimadzu, Kyoto, Japan) was used for analysis.

The total amount of soluble sugar was measured following previously described methods [46–48] with modifications. 5 mg of freeze-dried sample was used. Disrupting pigments were discarded using 100% acetone and soluble sugar was extracted using 0.5 mL of 80% ethanol. 0.5 mL of each sugar extract was reacted with 0.5 mL of ice-cold anthrone (Daejung, Korea) in 72% sulfuric acid (2 mg/mL) at 100 °C for 11 min, and the absorbance at 630 nm was measured.

### Artificial Diet

The semi-artificial diet was prepared by 3.33% agar solution with 5% freeze-dried *E. annuus* stem powder. The total amount of supplemented sugars and amino acids were determined by the observed difference between girdled upper part and non-girdled upper part of the stem using GC-MS. In addition, diets comprising double the amounts of supplements were prepared. The composition of supplemented nutrients in the diet was determined according to the data acquired from GC-MS (Fig. 4g): 37.97 mg/g galactose, 2.93 mg/g sucrose, 13.45 mg/g fructose, 0.11 mg/g valine, and 1.10 mg/g proline. Each larva was posited in an Eppendorf tube with a block of artificial diet. Every 3 days, larval mass was measured and a fresh diet was provided.

### Phytohormone quantification

The upper and lower parts (each 15 cm) of the girdled stem were collected one wpg and three wpg, respectively, when the larval feeding at each part was occurring. For the decapitated stems, only lower parts were sampled. To exclude bias originating from different water contents, the upper part samples were freeze-dried. Phytohormones were quantified according to the previously described method [49], with modifications [16]. One milliliter of ethyl acetate spiked with internal standards (20 ng each of d_4_-salicylic acid, d_5_-jasmonic acid, and d_6_-abscisic acid) was added to 10 mg of each sample. The solvent was evaporated using a centrifugal vacuum concentrator VC2124 (LaboGene, South Korea) at 30 °C and resuspended in 70% methanol and centrifuged at 13,000 rpm for 10 min. 200 µL of the supernatant was collected.

The phytohormones were measured using LC-MS. The extract was injected into a C18 column (UPLC BEH, 1.7 μm particle size, 100 mm length × 2.1 mm inner diameter, Waters, Ireland) and separated using a high-performance liquid chromatography system (Shimadzu, LCMS-8050). Solvent A consisted of deionized water containing 0.1% (v/v) acetonitrile and 0.05% formic acid. Solvent B consisted of 100% MeOH. The following gradient conditions were used for chromatography: 0.01–0.5 min 5% B; 0.6–6.7 min linear gradient 50–95% B; 6.7–8.7 min 95% B; and re-equilibration at 5% B for 1 min. The flow rate was set at 400 µL/min. The mass spectrometry conditions were as follows: capillary temperature, 300 °C; capillary voltage, 3.0 kV.

### Protease inhibitor assay

The upper lower part (15 cm) of the girdled stem was collected one wpg for protease inhibitor activity measurement. The procedure used here was modified from previous studies [50, 51]. Protein extraction was performed by adding 180 µL of extraction buffer (50 mM phosphate pH 7.2, 150 mM NaCl, and 2.0 mM EDTA) to 45 mg of each sample.

The protease activity of papain with the extracted protein was measured for each sample. The papain solution was prepared as 50 µL/mL papain in 25 mM sodium phosphate buffer (pH 7.0). 0.05 mL of papain solution, 0.1 mL of protein extract, and 0.1 mL of incubation buffer (0.25 M sodium phosphate buffer, pH 6.0, 2.5 mM EDTA, and 25 mM 2-mercaptoethanol) were mixed. After 5 min of incubation at 37 °C, the protease reaction was initiated by adding 0.1 mL of 1 mM Nα-benzoyl-DL-arginine β-naphthylamide hydrochloride (BANA) solution as the substrate. The reaction was terminated by adding 0.5 mL of 2% HCl in ethanol after 10 min of incubation at 37 °C. For color development, 0.5 mL of 0.06% p-dimethylaminocinnamaldehyde/ethanol was added. The resulting absorbance was measured at 540 nm as an indicator of protease activity and normalized by the fresh mass of the samples and the amount of total protein, which was measured using the BCA assay (Thermo Scientific kit 23,227, Waltham, MA, USA).

### Larval gut RNA extraction and RNA-sequencing

Larval gut RNA was extracted from *P. rufiventris* and *A. amurensis*. Three, one, and three biological replicates were sequenced for *P. rufiventris* reared in girdled stems, ungirdled stems, and *A. amurensis* larvae, respectively. The larvae were sterilized with 70% ethanol, rinsed with distilled water, and ice-cooled. The midguts were collected, rinsed with Ringer’s solution, and frozen in liquid nitrogen for grinding by pestle. The RNA was extracted using RNeasy Micro Kit (QIAGEN, Germany) and purified by RNeasy MinElute Cleanup Kit (QIAGEN). The library preparation was conducted using TruSeq Stranded mRNA LT Sample Prep Kit. Illumina paired-end 151 bp platform was used for sequencing.

### De novo transcriptome assembly

The quality of the raw reads was assessed using FastQC [52] (v. 0.11.8), and the reads were trimmed using Trimmomatic [53] (v 0.38). To remove contamination of plant-driven RNA, Kraken2 [54] (v. 2.1.2) was used to classify reads belonging to the plantdb. Processed reads were assembled using Trinity [55] (v. 2.10.0). Open reading frames were predicted using TransDecoder (v. 5.5.0; https://github.com/TransDecoder/TransDecoder) and the extracted sequences were annotated using Trinotate [56] (v. 3.2.2).

### Gene search

The Pfam annotations in Trinotate was used to initially search putative CYP450s, UGTs, COEs, GSTs, and ABC transporters in *de novo* assembled transcriptome of *P. rufiventris* and *A. amurensis*. The blastx search to SWISS-PROT was used to confirm predicted genes.

### Agapanthia amurensis choice assay

The oviposition preference of *Agapanthia amurensis* for girdled and non-girdled *E. annuus* plants was investigated. One week after experimental girdling, two girdled and two non-girdled *E. annuus* plants were placed symmetrically in a tent (47 × 47 × 91 cm^3^) in an open field site at Cheongju_1 (Supplementary Table S1). Each plant was placed 30 cm from the release point of a female *A. amurensis.* Each female was used for the choice assay within one month post emergence. *E. annuus* plants were exchanged once an oviposition choice was made and each choice was recorded.

### Co-infestation assay

The effect of the *A. amurensis* larva on *P. rufiventris* larva was investigated by inoculating an egg of *A. amurensis* into the bottom part of the *E. annuus* stem 3 d after *P. rufiventris* egg inoculation. Four weeks after egg inoculation, *E. annuus* stems were dissected to measure the survival rate of two larvae.

### Statistical analysis

All statistical analyses were conducted using the R software [57] (v. 4.0.5). The normality of raw data was checked using the Shapiro-Wilk test and similar variances between groups were tested using Levene’s test. For parametric tests, Student’s t-test and one-way ANOVA followed by Tukey’s honestly significant difference (HSD) test were used. For non-parametric tests, the Mann-Whitney U test and Kruskal-Wallis’s test followed by Dunn’s test were used.

(h) Relative growth rates (RGR) of *P. rufiventris* larva at 12 days reared with artificial diets containing supplementary sugars and amino acids (n.s., no significant differences, One-way ANOVA).

### Electronic supplementary material

Below is the link to the electronic supplementary material.

Supplementary Material 1

Supplementary Material 2

Supplementary Material 3

Supplementary Material 4

## Data Availability

The raw sequence files are deposited at NCBI (Bioproject: PRJNA1008891). All data supporting the results are available at Dryad (https://datadryad.org/stash/share/IyxFJF58UWR6tAdwLnlvsSqD4e4tC0UCS4IpXrv_wRY).

## Code availability

Codes used for bioinformatic analyses are deposited in Github (https://github.com/Phytoecia/Girdling_transcriptomics).

## Abbreviations

JA: jasmonic acid
GO: gene ontology
GC-MS: gas chromatography-mass spectrometry
CYP450: cytochrome P450
UGT: UDP-glycosyltransferase
GST: glutathione-S-transferase
COE: carboxylesterase
