# Supplementary material for: Girdling behavior of the longhorn beetle modulates the host plant to enhance larval performance
Source: BMC Ecol Evol. 2024 Apr 18;24:49. doi: 10.1186/s12862-024-02228-z (PMC11025245; doi:10.1186/s12862-024-02228-z)
Supplement: Supplementary file 2 — Supplementary Material 2 [file 12862_2024_2228_MOESM2_ESM.docx]

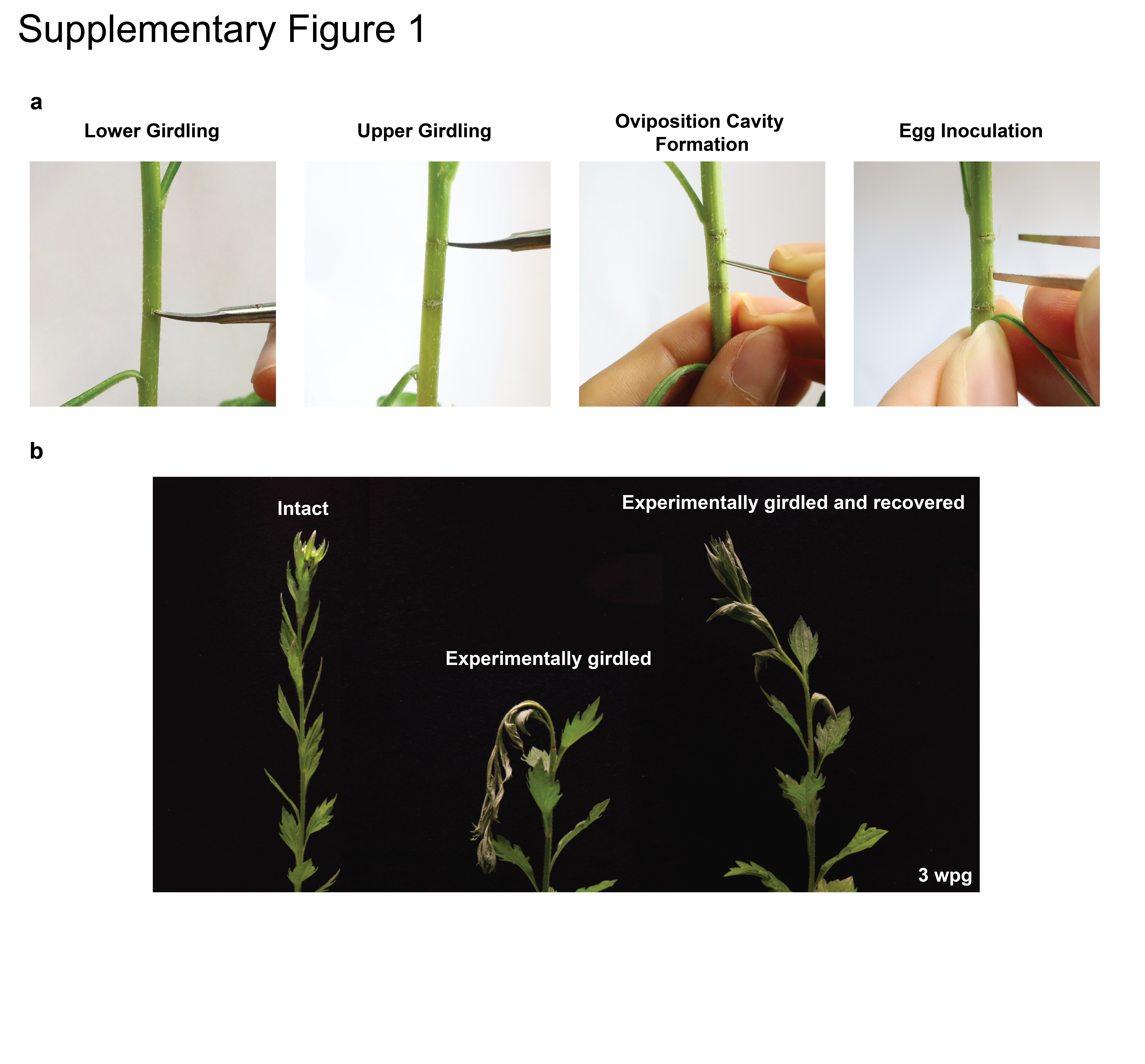


Supplementary Fig 1. Experimental girdling

1. Procedure of experimental girdling and egg inoculation
2. Morphology of intact, experimentally girdled, and recovered E. annuus plants 2 days post girdling.


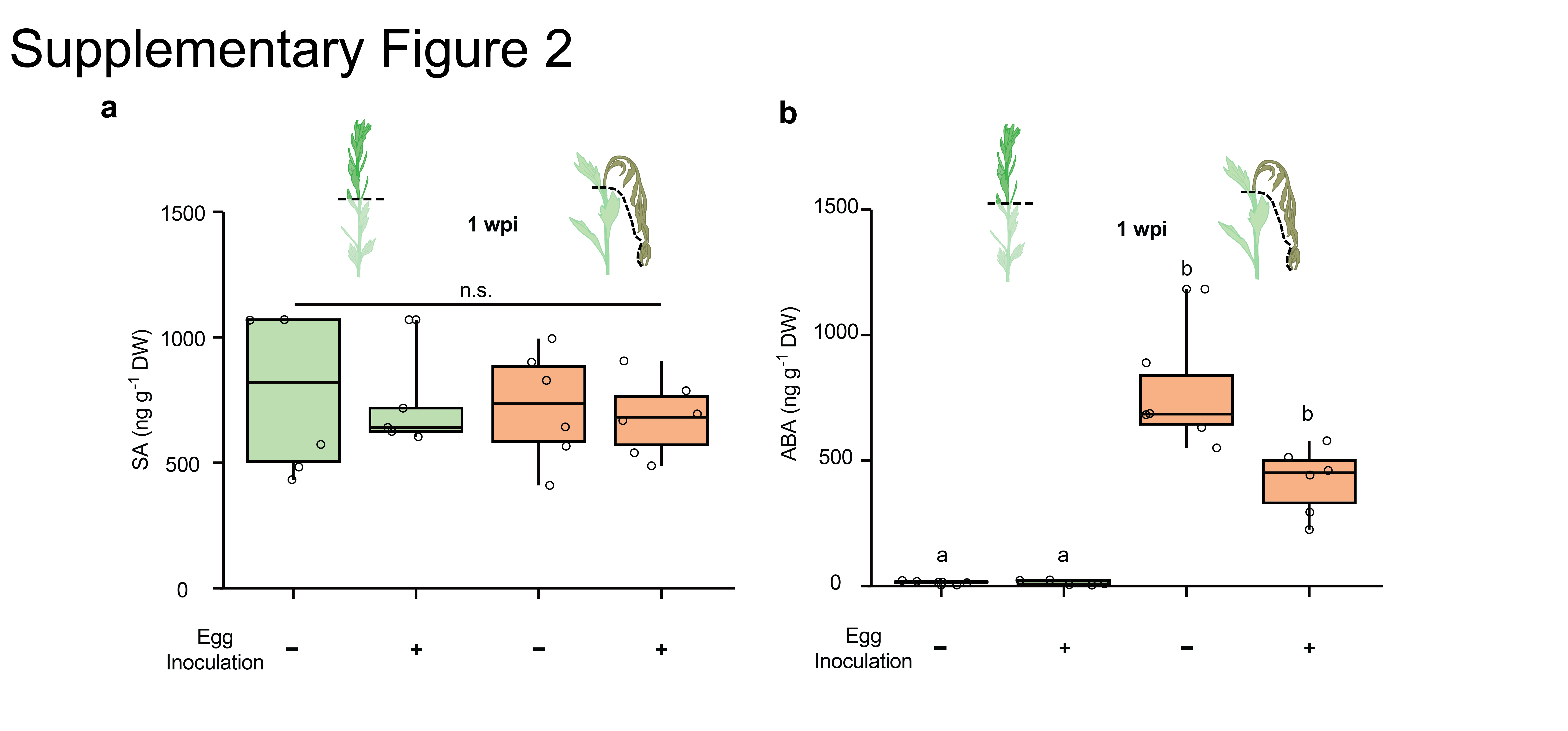


Supplementary Fig 2. Level of (a) salicylic acid and (b) abscisic acid measured at the upper part of the experimentally girdled and non-girdled stem of *E. annuus*. (n.s. no significant differences; different letters indicate significant differences, One-way ANOVA followed by Tukey’s HSD)


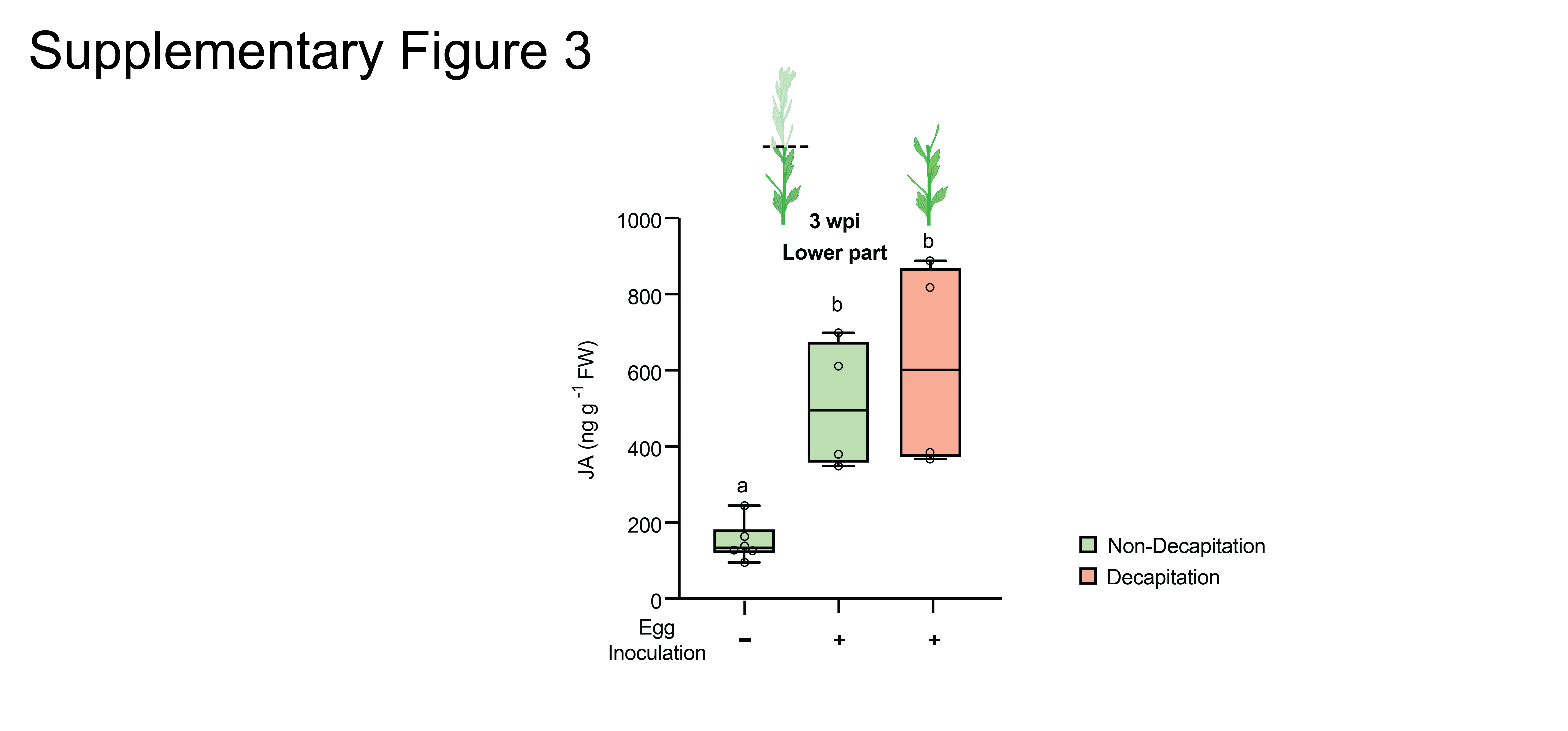


Supplementary Fig 3. JA contents in decapitated *E. annuus* stem upon *P. rufiventris* larval attack. Significant differences are indicated using different letters; *p* < 0.05; one-way ANOVA followed by *Tukey*’s HSD.


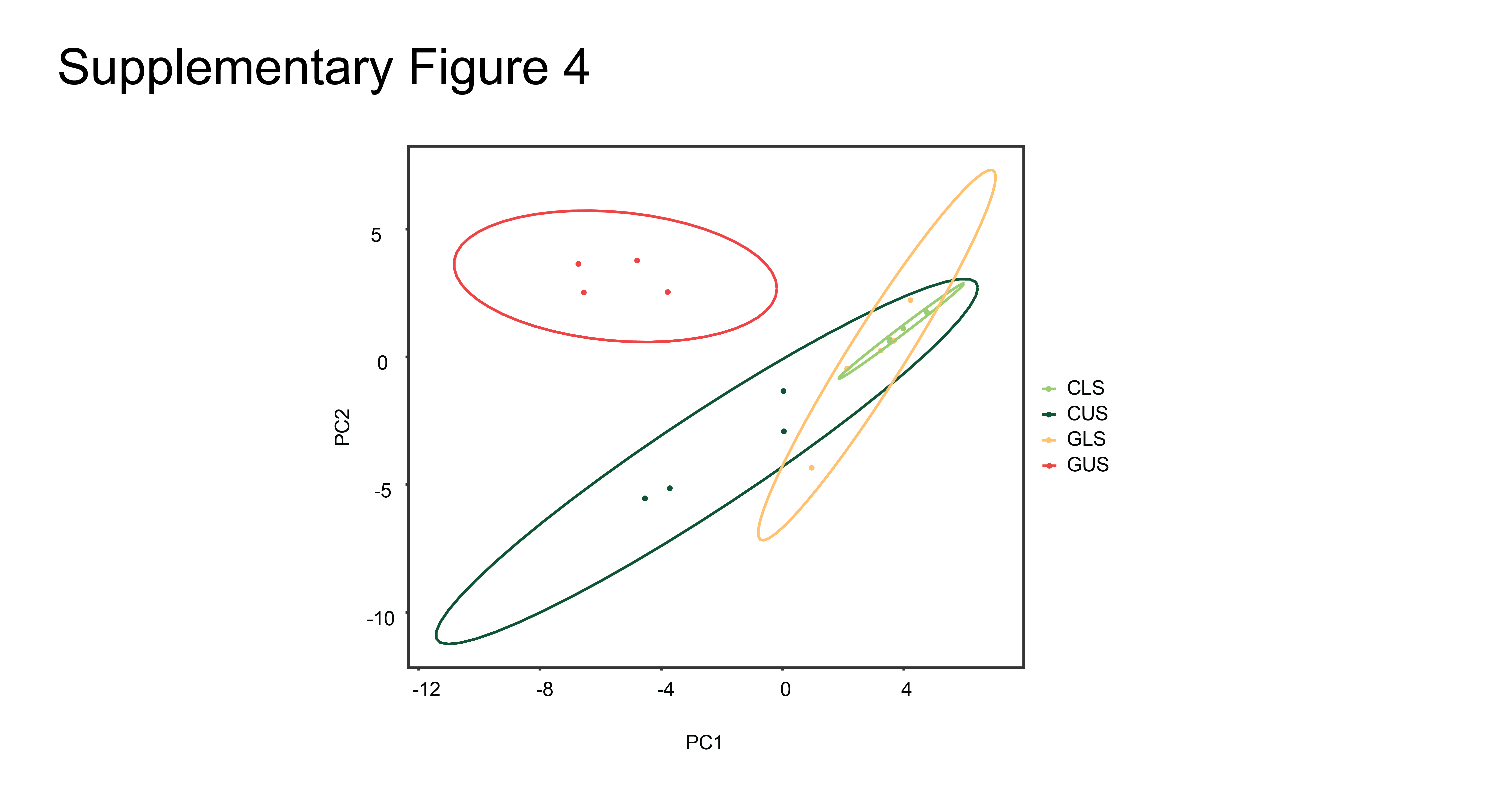


Supplementary Fig 4. Principal component analysis of the metabolic profiles of girdled and non-girdled stem of *E. annuus*. CLS, control lower stem; CUS, control upper stem; GLS, girdled lower stem; GUS, girdled upper stem.


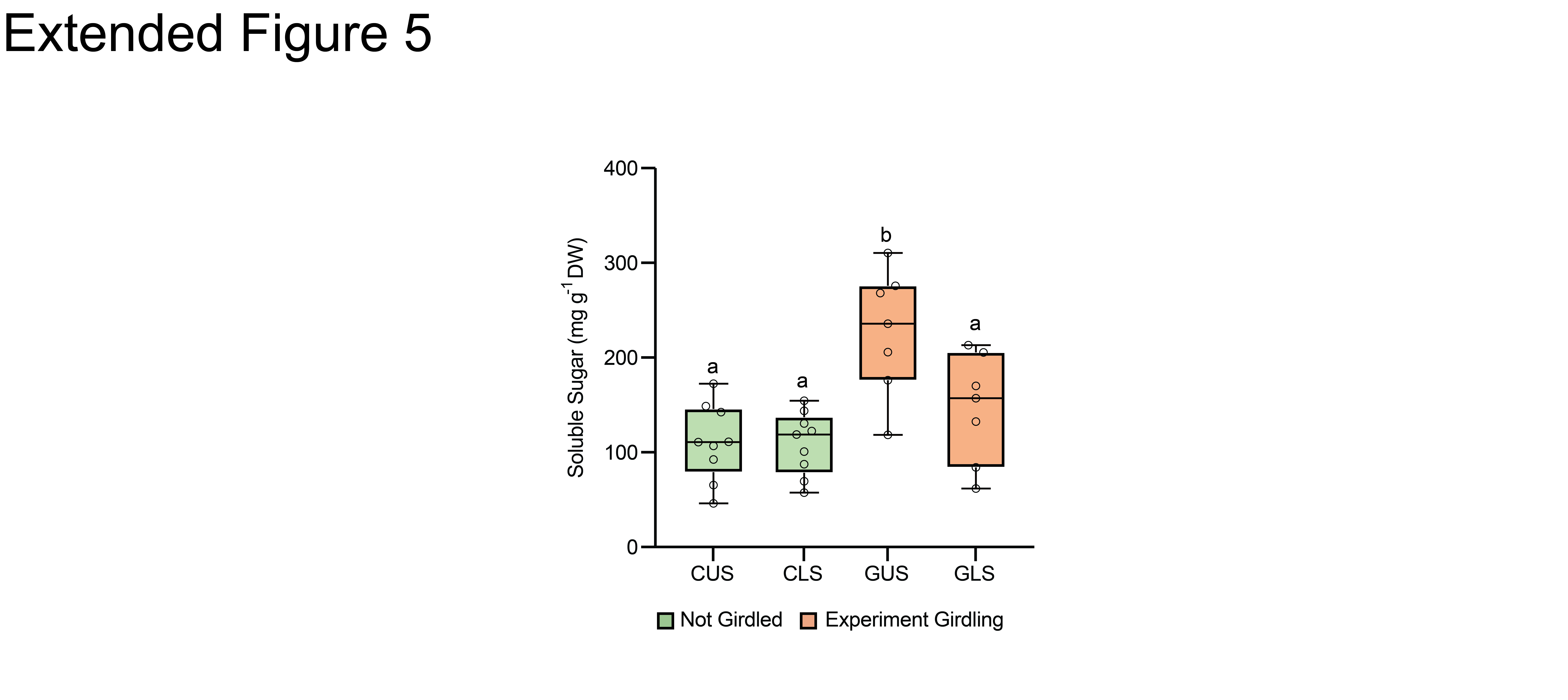


Supplementary Fig 5. Total soluble sugar of upper and lower stem of *E. annuus* with and without experimental girdling. Boxes indicate the 1^st^ and 3^rd^ quantiles (significant differences are indicated as different alphabets; p < 0.05; one-way ANOVA followed by Tukey’s HSD). CLS, control lower stem; CUS, control upper stem; GLS, girdled lower stem; GUS, girdled upper stem.


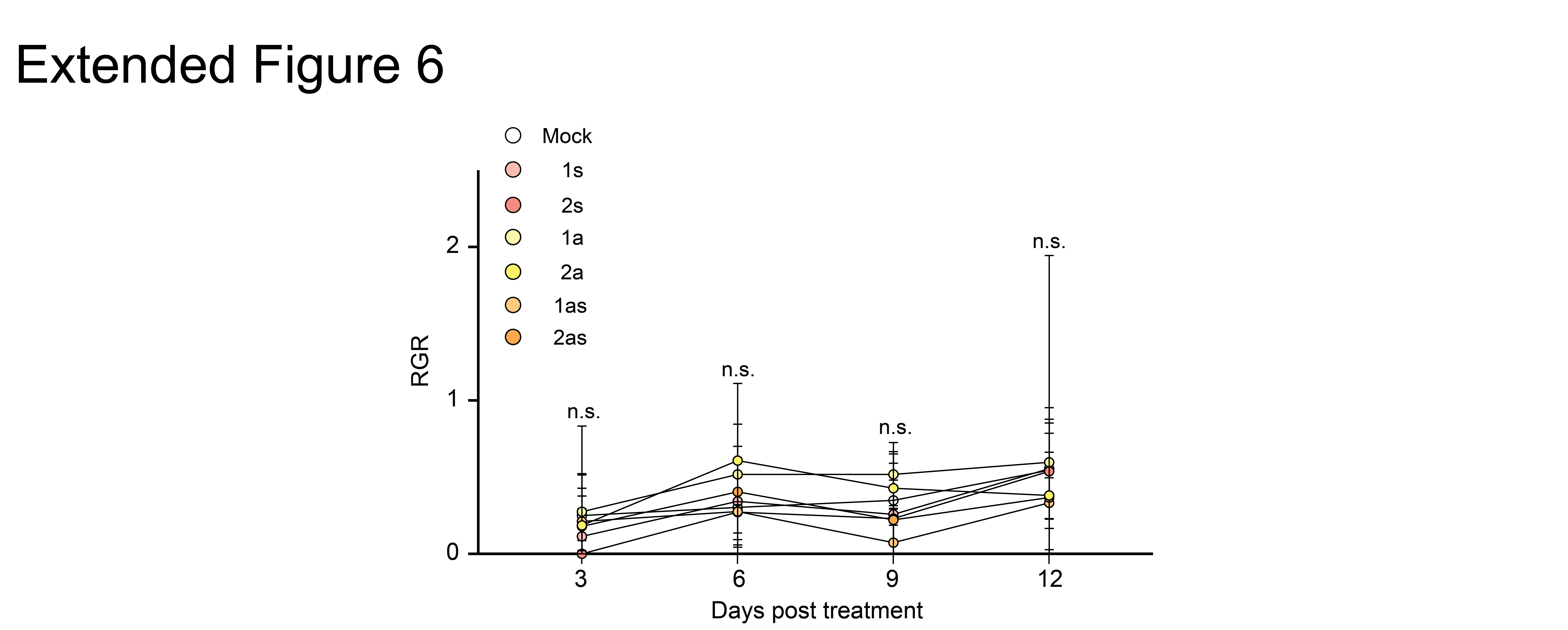


Supplementary Fig 6. Relative growth rate (RGR) of *P. rufiventris* beetles reared with artificial diets with and without sugar and amino acid supplementation. Error bars indicate standard errors (n.s., no significant differences; one-way ANOVA). Mock, no nutrient supplementation; 1s, 1x sugar; 2s, 2x sugar; 1a, 1x amino acids; 2a, 2x amino acids; 1as, 1x sugar and amino acids; 2as, 2x sugar and amino acids
